# Anti-Type VII Collagen Antibodies Are Identified in a Subpopulation of Bullous Pemphigoid Patients With Relapse

**DOI:** 10.3389/fimmu.2018.00570

**Published:** 2018-03-21

**Authors:** Delphine Giusti, Grégory Gatouillat, Sébastien Le Jan, Julie Plée, Philippe Bernard, Frank Antonicelli, Bach-Nga Pham

**Affiliations:** ^1^Laboratory of Dermatology, Faculty of Medicine, University of Reims Champagne-Ardenne, Reims, France; ^2^Laboratory of Immunology, Reims University Hospital, University of Reims Champagne-Ardenne, Reims, France; ^3^Department of Dermatology, Reims University Hospital, University of Reims Champagne-Ardenne, Reims, France; ^4^Department of Biological Sciences, Immunology, Faculty of Odontology, University of Reims Champagne-Ardenne, Reims, France

**Keywords:** anti-type VII collagen antibodies, bullous pemphigoid, epitope spreading, mucous membrane involvement, relapse

## Abstract

Bullous pemphigoid (BP) is an autoimmune bullous skin disease characterized by anti-BP180 and anti-BP230 autoantibodies (AAbs). Mucous membrane involvement is an uncommon clinical feature of BP which may evoke epidermolysis bullosa acquisita, another skin autoimmune disease characterized by anti-type VII collagen AAbs. We therefore evaluated the presence of anti-type VII collagen AAbs in the serum of BP patients with and without mucosal lesions at time of diagnosis and under therapy. Anti-BP180, anti-BP230, and anti-type VII collagen AAbs were measured by ELISA in the serum of unselected patients fulfilling clinical and histo/immunopathological BP criteria at baseline (*n* = 71) and at time of relapse (*n* = 24). At baseline, anti-type VII collagen AAbs were detected in 2 out of 24 patients with BP presenting with mucosal involvement, but not in patients without mucosal lesions (*n* = 47). At the time of relapse, 10 out of 24 BP patients either displayed a significant induction or increase of concentrations of anti-type VII collagen AAbs (*P* < 0.01), independently of mucosal involvement. Those 10 relapsing BP patients were also characterized by a sustained high concentration of anti-BP180 AAb, whereas the serum anti-BP230 AAb concentrations did not vary in BP patients with relapse according to the presence of anti-type VII collagen AAbs. Thus, our study showed that anti-type VII collagen along with anti-BP180 AAbs detection stratified BP patients at time of relapse, illustrating a still dysregulated immune response that could reflect a potential epitope spreading mechanism in those BP patients.

## Introduction

Bullous pemphigoid (BP) is the most common subepidermal autoimmune blistering skin disease that preferentially affects the elderly with various clinical manifestations. Clinically, BP patients typically present at diagnosis vesicles and tense clear blisters, which mainly occur on erythematous skin, together with erythematous or urticarial papules and plaques (1–4). BP is immunologically characterized by tissue-bound and circulating autoantibodies (AAbs) directed against either the BP antigen 180 (BP180) or the BP antigen 230 (BP230) or even both, which are components of hemidesmosomes involved in the dermal–epidermal cohesion (2, 3, 5–9). Most of BP patients (82–94%) display in their serum AAb that bind to the NC16A domain of the transmembrane protein BP180 (3, 10–16). AAbs against other antigenic sites of BP180 are associated with the severity and the phenotype of BP. Depending on the detection assay used, the presence of AAb against both the BP180 N- and C-terminal of the ectodomain was found to be associated with the presence of mucosal lesions (12), or not (17). When presenting with mucosal involvement, BP may suggest the BP-like, inflammatory form of epidermolysis bullosa acquisita (EBA), another skin autoimmune disease characterized by AAb directed against type VII collagen, a protein of the basement membrane zone beneath the stratified squamous epithelia (8, 18–22). However, no study has investigated yet the presence of serum anti-type VII collagen AAb at time of diagnosis of BP patients with mucosal involvement and whether these AAb are associated with BP outcome.

Loss of immune self-tolerance eventually leads to the generation of AAb (22). Among the different AAb in BP, the pathogenicity was mainly attributed to those directed against the NC16A domain of BP180 (9, 10, 23, 24). Actually, high serum level of anti-BP180 NC16A AAb was correlated with disease activity at time of diagnosis and was shown as an independent risk factor for BP relapse after cessation of therapy (14, 15, 25–27). In addition, approximately 30% of the BP patients relapse during the first year of treatment (16, 28–31). For those latter BP patients, it was shown that the variations of serum IgG AAb directed toward the BP180 NC16A domain after 2 months of therapy may be useful to predict BP outcome (16, 32). However up to now, a comparison in BP patients of the AAb profile at time of relapse vs. baseline has been investigated neither with respect to mucosal involvement nor with respect to antibody directed against other epidermal basement membrane autoantigens such as the type VII collagen.

In this study, we investigated certain autoimmunity markers which result from the disturbance of self-tolerance in BP both at baseline with respect to mucous membrane involvement and at time of relapse. To that purpose, the presence of AAb against type VII collagen was evaluated at time of diagnosis both in the serum of BP patients with and without mucosal involvement. Furthermore, serum anti-type VII collagen AAb titer at baseline and at time of relapse was analyzed according to the initial clinical BP phenotype at baseline and compared with anti-BP180 and anti-BP230 AAb serum profiles.

## Materials and Methods

### Patients

This retrospective study was conducted in the Dermatology Department of the University Hospital of Reims, belonging to the French Reference Center for Autoimmune Bullous Diseases. From January 2011 to July 2015, 71 consecutive patients with newly diagnosed BP were included in this study when they fulfilled the following criteria: (1) blistering skin dermatosis fulfilling at least three of four clinical criteria for BP (33) and (2) linear IgG and/or C3 deposits along the epidermal basement membrane zone by skin direct immunofluorescence microscopy of perilesional skin. Patients fulfilling less than three clinical criteria, were also included if they demonstrated high anti-BP180/230 titers and at least roof labeling by indirect immunofluorescence (IIF) on salt split skin (SSS). During a 1-year follow-up, the number and dates of potential relapses were recorded. Relapse was defined as the reappearance of at least three daily new blisters along with pruriginous, erythematous, or urticarial plaques (34). The disease activity was assessed at baseline using the Bullous Pemphigoid Disease Area Index (BPDAI) (34). Patients were separated into two groups and then analyzed according to the presence or the absence of mucosal involvement as recorded in the BPDAI evaluation.

### AAbs Detection

For each patient, blood samples were collected in clot activator tubes. Sample collection was realized at the time of diagnosis in all BP patients, and at the time of relapse on therapy during the follow-up (mean time of 39 weeks after the beginning of treatment), or at an equivalent follow-up visit for patients with ongoing remission. All sera were stored at −20°C until analysis. The detection of serum anti-type VII collagen AAb was performed with a commercially available ELISA (MBL, Nagoya, Japan). This assay uses the NC1 and NC2 domains of type VII collagen as substrates (35). The 6 U/mL cutoff value recommended by the manufacturer was used. Anti-BP180-NC16A and anti-BP230 AAb were detected in serum samples using commercially available ELISA tests (MBL, Nagoya, Japan) (36, 37). ELISA values are expressed as Units per milliliter of serum with the cutoff value of 9 U/mL for both ELISAs as recommended by the manufacturer. Serum anti-basement membrane zone AAb were detected by IIF on monkey SSS, according to the manufacturer’s instructions (Euroimmun, Lübeck, Germany).

### Statistical Analysis

Quantitative variables were described as mean ± SD and qualitative data as number and percentage. Comparisons between groups were performed using Wilcoxon rank test, Fisher exact test or χ^2^ test, as appropriate. A *P*-value <0.05 was considered statistically significant. All analyses were performed using Microsoft Excel and GraphPad Prism software.

## Results

### Clinical and Immunological Characteristics of BP Patients According to Mucosal Involvement at Baseline

To investigate the potential implication of serum anti-type VII collagen AAb at the time of diagnosis and during follow-up on therapy in patients with BP, 71 patients were included in the study. Clinical and immunological characteristics of BP patients according to mucosal involvement at baseline are detailed in Table 1. Among the 71 BP patients, 47 (66%) had a typical clinical presentation and 24 (34%) also had mucous membrane involvement, i.e., blisters or erosions of the oral cavity in all 24 cases in addition with genital erosions in 1 case. We evidenced that the presence of oral lesions was associated with higher total (Table 1, *P* < 0.001) and skin BPDAI scores (*P* < 0.01). We also found that anti-BP180 AAb were indifferently present in BP patients with or without mucosal lesions, with similar titers (Table 1), while the percentage of patients with positive anti-BP230 antibodies titer was lower in patients with mucosal involvement compared with patients without mucosal involvement (29 and 55%, respectively; *P* < 0.05). Further investigation of AAb expression in BP patients with and without mucosal involvement showed that, although not statistically significant, anti-type VII collagen AAb were only detected at diagnosis in the serum of patients presenting mucosal lesions (2/24 patients with mucosal involvement vs. 0/47 in patients without mucosal involvement, Table 1). In those two patients, the presence of high titers of serum anti-BP180 (66 and 109 U/mL) but low titers of anti-type VII collagen (13 and 8 U/mL) AAb confirmed the diagnosis of BP.

**Table 1:** Clinical and immunological characteristics of BP patients at baseline.

	Total	Patients without mucosal involvement	Patients with mucosal involvement	*P* value
Patients no.	71	47	24	
Mean age ± SD (range), years	80.3 ± 10.1 (45 – 95)	82.6 ± 7.9 (53 – 95)	75.8 ± 12.6 (45 – 92)	0.05
Sex ratio (female/male)	1.7	2.1	1.2	0.25
Total BPDAI (mean ± SD)	46.0 ± 27.4	38.0 ± 22.9	61.3 ± 29.7	0.001
Skin BPDAI (mean ± SD)	44.8 ± 26.1	38.0 ± 22.9	57.9 ± 28.2	0.004
Relapse, patients no. (%)	24 (33.8)	13 (27.6)	11 (45.8)	0.12
**Serum autoantibodies**				
COL7 Ab				
Positive ELISA value, No (%)	2 (2.8)	0 (0.0)	2 (8.3)	0.11
Mean ± SD (U/mL)	2.6 ± 1.8	2.4 ± 1.3	3.1 ± 2.6	0.29
BP180 Ab				
Positive ELISA value, no. (%)	59 (83.1)	40 (85.1)	19 (79.2)	0.52
Mean ± SD (U/mL)	67.1 ± 50.0	65.8 ± 48.6	69.5 ± 56.2	0.80
BP230 Ab				
Positive ELISA value, no. (%)	33 (46.5)	26 (55.3)	7 (29.2)	0.046
Mean ± SD (U/mL)	26.2 ± 36.9	31.4 (39.7)	16.1 ± 28.8	0.10
IIF-SSS, no. (%)				
Roof labeling	47 (66.2)	33 (70.3)	14 (58.3)	
Roof and floor labeling	4 (5.6)	3 (6.4)	1 (4.2)	
Floor labeling	0 (0.0)	0 (0.0)	0 (0.0)	
Negative	20 (28.2)	11 (23.4)	9 (37.5)	

*Clinical and immunological characteristics at baseline from the whole population and comparison of these characteristics between BP patients with and without mucosal involvement*.

*BPDAI, Bullous Pemphigoid Disease Area Index; COL7 Ab, anti-type VII collagen autoantibodies; BP180 Ab, anti-BP180 antibodies; BP230 Ab, anti-BP230 antibodies; IIF-SSS, indirect immunofluorescence on salt split skin; BP, bullous pemphigoid*.

### Clinical and Immunological Characteristics of BP Patients With Relapse

**Figure 1:**
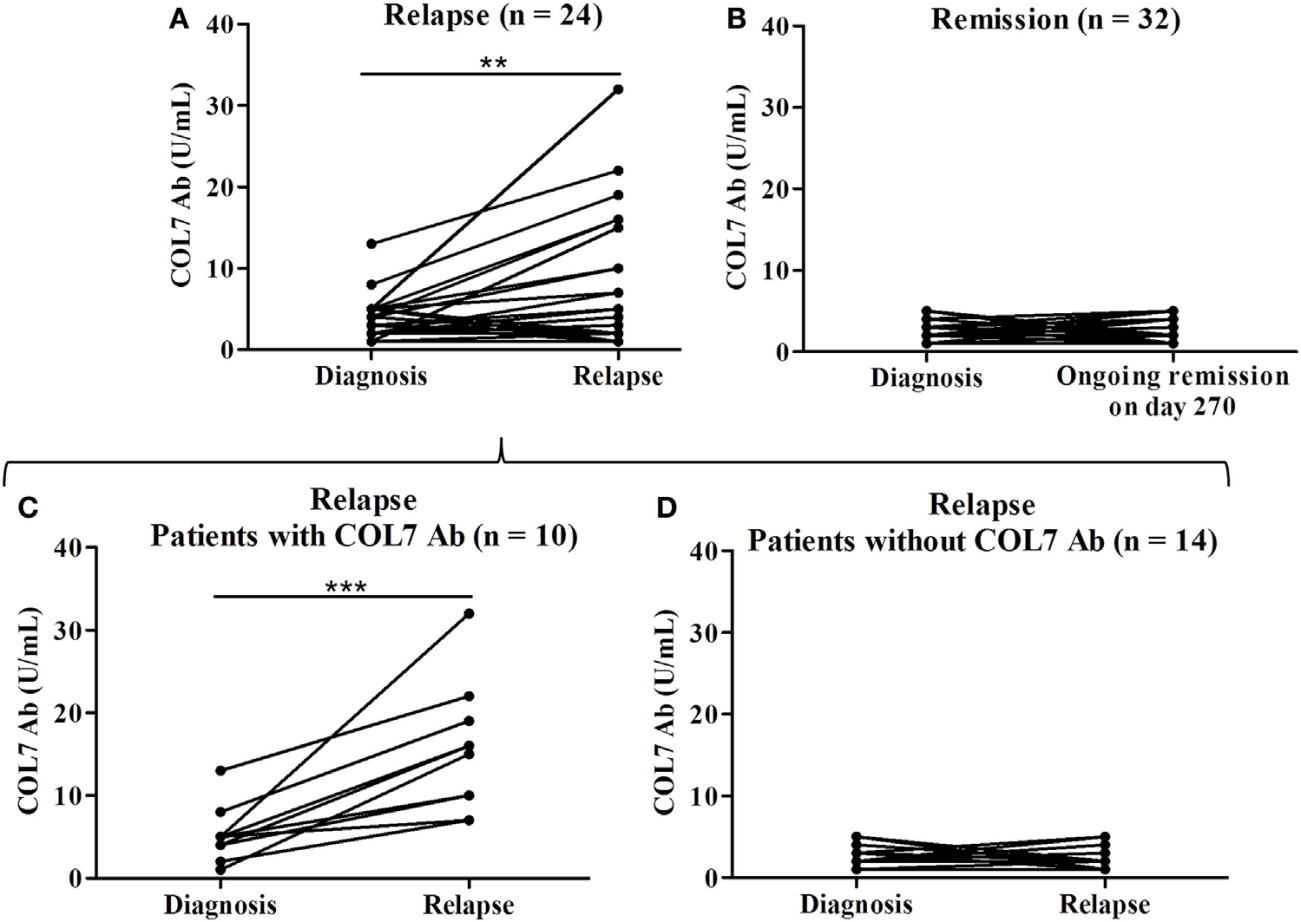
Serum levels of anti-type VII collagen AAb (COL7 Ab) in patients with relapse (*n* = 24) (mean value at time of relapse 7.8 U/mL vs. mean value at baseline 3.6 U/mL) **(A)** and in patients with ongoing remission (*n* = 32) **(B)**. Mean delay between diagnosis and relapse was 270 days. Among the 24 patients with relapse, 10 patients were identified with positive ELISA score of anti-type VII collagen AAb at time of relapse (mean value at time of relapse 15.4 U/mL) **(C)** and 14 patients experienced relapse without presenting anti-type VII collagen AAb **(D)**. Comparison was made either between baseline and relapse or between baseline and day 270 for patients with ongoing remission (**P < 0.01, ***P < 0.001).

Of the 71 BP patients included in this study, 56 had both a complete clinical and biological follow-up of at least 1 year, including BPDAI scores and serum AAb detection every 2 months and also in case of relapse. At baseline, 24 out of those 56 BP patients experienced at least one relapse during this follow-up period. Both total and skin BPDAI scores tended to be higher for BP patients who relapsed than for BP patients who remained in clinical remission upon therapy (Table 2). Mucosal involvement at time of diagnosis was not more frequent in patients with later relapse than in BP patients with ongoing remission (46 and 31%, respectively; *P* = 0.26). For those 24 BP patients, the relapse occurred in a mean delay of 39 weeks after starting treatment, without any difference between patients with and without mucosal involvement at baseline (Table 2). At the time of relapse, the level of serum anti-type VII collagen AAb was higher compared with baseline values (mean value at relapse 7.8 U/mL vs. mean value at baseline 3.6 U/mL, *P* < 0.01) (Figure 1A). Such an increase was not observed in the serum of BP patients with ongoing remission when analyzed after a similar median duration of treatment (Figure 1B).

**Table 2:** Clinical characteristics of BP patients at baseline according to disease outcome.

	Ongoing remission	Relapse	*P* value
Patients no.	32	24	
Mean age ± SD (range), years	77.9 ± 10.3 (53 – 90)	80.8 ± 11.5 (45 – 95)	0.19
Sex ratio (female/male)	1.66	1.67	1.00
Total BPDAI (mean ± SD)	42.3 ± 28.8	54.4 ± 25.0	0.08
Skin BPDAI (mean ± SD)	40.3 ± 27.5	52.6 ± 24.9	0.06
Mucosal involvement, no. (%)	10 (31.2)	11 (45.8)	0.26

*BPDAI, Bullous Pemphigoid Disease Area Index*.

### Anti-Type VII Collagen Antibody Positivity in a Subset of BP Patients at Time of Relapse

**Figure 2:**
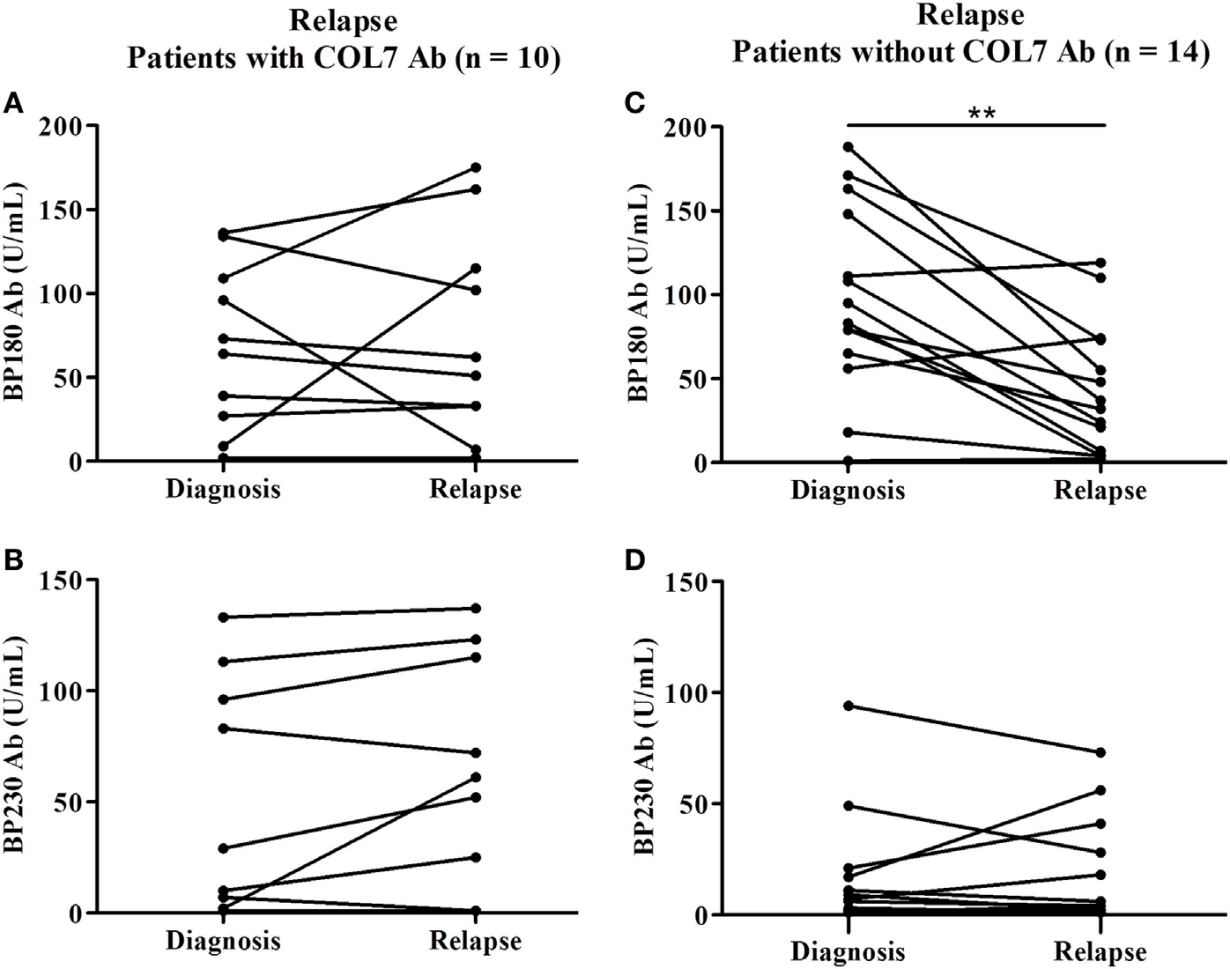
Serum levels of anti-BP180 (BP180 Ab), anti-BP230 (BP230 Ab) autoantibodies (AAbs) in the relapsing patients (*n* = 24). Comparison was made between baseline and relapse either in patients with increased concentrations of anti-type VII collagen AAbs **(A,B)** or in patients without detectable anti-type VII collagen AAbs **(C,D)** (**P < 0.01).

We then attempted to further characterize the subset of BP patients who relapsed during the follow-up. Serological analysis revealed that 10 (41%) of those 24 BP patients had increased serum levels of anti-type VII collagen AAb at time of relapse (mean value at time of relapse 15.4 U/mL) (Figure 1C), whereas the other relapsing patients did not (Figure 1D). Among those 10 BP patients with detectable serum anti-type VII collagen AAb at time of relapse, 6 had mucosal involvement at time of diagnosis. Other clinical features at baseline, including mean age, sex ratio, and BPDAI scores, were not different in patients with positive anti-type VII collagen at time of relapse from those who did not express this latter AAb (Table 3). Conversely, biological investigation of serum anti-BP180/230 AAb profiles between baseline and relapse highlighted that anti-BP180 ELISA scores remained elevated the 10 BP patients with concomitant positive anti-type VII collagen AAb, while they decreased in the subgroup of BP patients without anti-type VII collagen antibodies (Figures 2A,C). Such a difference in the serum AAb profiles was not observed when analyzing the variations in anti-BP230 antibody titers (Figures 2B,D).

**Table 3:** Clinical characteristics at baseline of BP patients who further relapsed under treatment.

	Patients with secondary appearance of COL7 Ab at relapse (*N* = 10)	Patients without secondary appearance COL7 Ab at relapse (*N* = 14)	*P* value
Mean age ± SD (range), years	79.8 ± 14.3 (45–95)	81.4 ± 9.6 (57.95)	1.00
Sex ratio (female/male)	2.5	1.3	0.52
Total BPDAI (mean ± SD)	57.3 ± 34.0	52.3 ± 17.0	0.72
Skin BPDAI (mean ± SD)	54.2 ± 32.4	51.5 ± 16.7	1.00
Mucosal involvement, no. (%)	6 (60.0)	5 (35.7)	0.24

*The clinical characteristics at baseline of BP patients who further relapsed under treatment were analyzed according to the secondary appearance of anti-type VII collagen autoantibodies at relapse (COL7 Ab)*.

*BPDAI, Bullous Pemphigoid Disease Area Index; BP, bullous pemphigoid*.

## Discussion

The present study demonstrated that investigating both anti-BP180 and anti-type VII collagen antibodies serum concentrations was useful to exclude EBA diagnosis in BP patients with mucosal involvement at time of diagnosis. Furthermore, anti-type VII collagen AAb were identified in a subgroup of BP patients at time of relapse.

At diagnosis, anti-type VII collagen antibodies were detected only in 2 of 24 BP patients with oral involvement. However, high serum anti-BP180 titers compared to those of anti-type VII collagen antibodies advocated for a BP rather than an EBA for which the presence of serum anti-type VII collagen antibody remains the immunological hallmark at baseline (20, 35, 38). Although serum anti-type VII collagen antibodies were detected in the subgroup of BP patients with mucosal involvement, such a low frequency of antibody expression cannot be considered as a biological marker of mucosal subepidermal blistering in BP. Of note, this very low frequency of BP patients with serum anti-type VII collagen AAb and their low titers are in accordance with previous studies evaluating the performance of anti-type VII collagen antibody ELISA, in which 1–8% of patients with BP were positively tested (35, 39, 40). This is also in setting with the low prevalence of anti-type VII collagen antibodies in other autoimmune and autoinflammatory diseases, such as inflammatory bowel disease (16%) and pemphigus (9.5%), but also in healthy subjects (38). Furthermore, the low titer of anti-type VII collagen AAb in comparison with the high titer of anti-BP180 antibodies in our two patients with mucous membrane involvement, like in these other diseases, rather points out the production of anti-type VII collagen antibodies in BP as an epiphenomenon, as previously suggested (31). A possible diversification of the AAb response in BP could be related at least in part to disease severity, as BP patients with mucosal involvement had higher total and skin BPDAI scores, especially the blisters/erosions activity score, as compared with the BPDAI score from patients with a typical form of BP (41).

Serum anti-type VII collagen antibodies were evidenced in about 40% of patients at the time of relapse and their titers were increasing. This is in line with a previous study which showed that the variation in the AAb profile, called epitope spreading, occurred as an early event in about 50% of BP patients (42). Our results complete our knowledge on BP-associated epitope spreading, by showing that the production of AAb against type VII collagen occurred in relapsing patients but not in patients with ongoing remission. This further illustrated that targets of immune responses in BP can be extended not only to other epitopes on the hemidesmosome protein (42, 43) but also to other proteins in their vicinity. The production of anti-type VII collagen AAb at time of relapse was not related to mucosal involvement at baseline, but to disease severity. Of note, it has been previously showed that the main predictive risk factor of relapse is the number of new daily blister at baseline (16). By contrast, the increase in the skin BPDAI score in BP patients with mucosal involvement was rather related to erosions than to blister formation. Thus, anti-type VII collagen production may result from situations in which tissue damage, induced by proteases activity linked to blister formation, causes the release and exposure of a previously encrypted antigen, thereby leading to a secondary autoimmune response against the newly exposed antigen as proposed for other autoimmune diseases (38, 39).

Noteworthy, serum anti-type VII collagen AAb was evidenced only in a subgroup of BP patients at the time of relapse. Then, the presence of anti-type VII collagen AAb may be an associated risk factor characterizing this subgroup of relapsing BP patients. Interestingly, this subgroup was also characterized by a high persistent titer of BP180 antibodies. Anti-BP180 antibody concentration has been correlated with BP disease activity (14, 15, 25, 26), and the decrease in anti-BP180 AAb levels after 2 months of treatment was lower in patients with further relapse in comparison with patients with ongoing remission (16). In the present study, if the serum level of anti-BP180 antibody remained elevated in all BP patients at time of relapse, we noticed that BP relapsing patients with serum anti-type VII collagen AAb displayed a sustained level of anti-BP180 antibody, suggesting that the immune response is still highly active in those patients. We also previously showed that the inflammatory response remained elevated in BP patients who relapsed during the first year of treatment. Especially, the production of IL-17, IL-23, CXCL10, and ECP also remained elevated after 2 months of treatment in the serum of relapsing BP patients (44–47). Then, one can hypothesize that the inflammatory response may also display a specific profile in this subgroup of BP patients with serum anti-type VII collagen AAb and high serum level of anti-BP180 at time of relapse. Of note, regulatory T (Treg) cells, a major regulatory system of autoimmunity, demonstrated plasticity and can convert to Th17 cells according to the cytokine environment (48). Accordingly, a previous study evidenced that Treg cells were upregulated (49), whereas another study proposed a Treg cell downregulation in BP (50). Knowing that Treg activity and IL-17-producing cells may have opposite effects on autoimmunity (51, 52), an impaired Treg activity in an IL-17/IL-23 context (53, 54) could result in an imbalance between the pro-inflammatory and the regulatory cytokines levels which control the tolerance breakdown limit and therefore favors the production of anti-type VII collagen AAb and the sustained concentration of anti-BP180 AAb (47, 53, 55–57). In this line, it is worth to note that all of these inflammatory molecules promote matrix metalloproteinase MMP-9 and neutrophil elastase production and therefore participate to tissue degradation (44, 45, 53). Although further investigations are still needed to explain why some BP patients had circulating anti-type VII collagen AAb at time of relapse and not the other relapsing patients, our results support the hypothesis that chronic and dysregulated inflammation in line with persistent tissue damage and exposure of autoantigens may lead to tolerance breakdown and to autoimmunity.

In conclusion, we here showed the presence of AAb against the type VII collagen in the serum of relapsing BP patients who had a severe and difficult to treat disease. Actually, this is the first study demonstrating in those relapsing patients that the immune response is still dysregulated, probably due to prolonged epidermal/dermal damages which may sustain the immune tolerance breakdown process. Based on this observation, it will be interesting in a future prospective study to evaluate whether the autoimmune response spreads to other autoantigens identified in the subepidermal autoimmune-mediated blistering diseases such as the laminin-332 and the laminin gamma-1 chain (58, 59). Furthermore, it will also be of interest to determine whether the presence of serum anti-type VII collagen antibodies could be a predictive factor for relapse by analyzing the concentration of these AAbs at an earlier time point during the patients’ follow-up in future prospective longitudinal studies. Finally, the pathogenicity of those anti-type VII collagen AAb in association with anti-BP180 NC16A AAb will also need to be investigated in animal models of BP (21, 60).

## Ethics Statement

This study was carried out in accordance with the recommendations of the “Commission Nationale de l’Informatique et des libertés (CNIL)” and approved by the Ethic Committee of the University Hospital of Reims (institutional review board 14-04-2009). All subjects gave written informed consent in accordance with the Declaration of Helsinki.

## Author Contributions

DG and GG wrote the main manuscript text. DG, GG, and SJ conducted the experiments and statistical analyses. PB and JP participated to patients’ care and clinical follow-up. FA, PB, and B-NP supervised this work and manuscript redaction. All the authors reviewed the article.

## Conflict of Interest Statement

The authors declare that the research was conducted in the absence of any commercial or financial relationships that could be construed as a potential conflict of interest.
